# Diagnostic accuracy of metagenomic next-generation sequencing in pulmonary tuberculosis: a systematic review and meta-analysis

**DOI:** 10.1186/s13643-024-02733-8

**Published:** 2024-12-27

**Authors:** Yajie You, Ying meng Ni, Guochao Shi

**Affiliations:** 1 Department of Transfusion, First Affiliated Hospital of Nanjing Medical University, Nanjing, China; 2 Department of Respiratory and Critical Care Medicine, Ruijin Hospital, Shanghai Jiao Tong University School of Medicine, Shanghai, China; 3 Shanghai Key Laboratory of Emergency Prevention, Diagnosis and Treatment of Respiratory Infectious Diseases, Shanghai, China

**Keywords:** Diagnosis, Metagenomic next-generation sequencing (mNGS), Xpert-MTB/RIF, Pulmonary tuberculosis (PTB), Bronchoalveolar lavage fluid (BALF)

## Abstract

**Background:**

Metagenomic next-generation sequencing (mNGS) has emerged as a promising tool in clinical practice due to its unbiased approach to pathogen detection. Its diagnostic performance in pulmonary tuberculosis (PTB), however, remains to be fully evaluated.

**Objective:**

This study aims to systematically review and Meta-analyze the diagnostic accuracy of mNGS in patients with PTB.

**Methods:**

We conducted a literature search in PubMed (MEDLINE), Web of Science, Cochrane, and EMBASE databases, including studies published up to 2024. Studies comparing the diagnostic accuracy of mNGS with other methods such as Xpert-MTB/RIF and Mycobacteria tuberculosis (MTB) culture using bronchoalveolar lavage fluid (BALF), sputum, and lung biopsy tissue were included. Preclinical studies, review articles, editorials, conference abstracts, and book chapters were excluded. Statistical analysis was performed using Rev-man5, R package metabias, and Stata software.

**Results:**

Thirteen studies met the inclusion criteria and were included in the meta-analysis. The pooled sensitivity and specificity of mNGS for PTB were 83% (95% CI: 69–91%) and 99% (95% CI: 92–100%), respectively. Subgroup analyses revealed that in BALF, mNGS demonstrated a pooled sensitivity of 73% (95% CI: 61–82%) and specificity of 98% (95% CI: 92–100%); in the sputum, the pooled sensitivity was 60% (95% CI: 38–87%) with a specificity of 99% (95% CI: 96–100%); and in the lung biopsy tissue, the pooled sensitivity was 71% (95% CI: 38–95%) and the specificity was 98% (95% CI: 93–100%).

For Xpert-MTB/RIF, the pooled sensitivity and specificity were 72% (95% CI: 53–85%) and 100% (95%CI: 100–100%), respectively. Subgroup analyses demonstrated that in BALF, Xpert-MTB/RIF exhibited a pooled sensitivity of 69% (95% CI: 53–81%) and a specificity of 100% (95% CI: 77–100%).

The pooled sensitivity and specificity of mycobacteria culture were 50% (95% CI: 36–64%) and 100% (95% CI: 83–100%), respectively. Subgroup analyses indicated that in BALF, the pooled sensitivity of mycobacteria culture was 44% (95% CI: 37–52%) with a specificity of 100% (95% CI: 8–100%); in the sputum, the pooled sensitivity was 42% (95% CI: 21–65%) and the specificity was 100% (95% CI: 100–100%).

When combining mNGS with Xpert-MTB/RIF, the pooled sensitivity and specificity were 79% (95% CI: 40–97%) and 98% (95% CI: 95–100%), respectively.

**Conclusion:**

mNGS demonstrates similar diagnostic accuracy to Xpert-MTB/RIF in PTB and outperforms mycobacteria culture in terms of sensitivity. Furthermore, mNGS exhibits good detection capabilities across various PTB clinical samples.

**Systematic review registration:**

PROSPERO CRD42023427586.

## Introduction

Tuberculosis (TB), an infectious disease that has plagued humankind for millennia, remains a significant global health challenge. In 2022, a staggering 7.5 million individuals were infected with TB, resulting in 1.3 million deaths worldwide, including those co-infected with HIV [1]. Despite the progress made through the “Global Plan to Stop TB” (2006–2015), TB remains the leading cause of death from a single infectious agent, surpassing HIV/AIDS and malaria. In response, the World Health Organization (WHO) launched the “The End TB Strategy” aiming to halt the global TB epidemic by 2035, with a targeted reduction in deaths by 95% and incidence by 90% compared to the levels in 2015 [2, 3].

A significant impediment to achieving these goals is the diagnostic challenge posed by TB. Notably, approximately 40% of estimated incident cases remain undiagnosed and unreported [4]. The diagnostic toolbox for tuberculosis encompasses methods such as acid-fast bacilli (AFB) smears, mycobacterial culture, GeneXpert-MTB/RIF assay, and T-SPOT.TB assay. While AFB tests offer a rapid diagnostic option, they suffer from limited sensitivity and potential confusion with nontuberculous mycobacteria (NTM) [5]. Mycobacterial culture, once considered as the “gold-standard” for TB diagnosis [6, 7], is time-consuming, prone to contamination, and requires further biochemical tests [8]. The T-SPOT.TB assay, a commonly used auxiliary test for TB diagnosis, can be influenced by factors such as age, BMI, and immune status [9, 10]. Xpert-MTB/RIF, a nucleic acid amplification test recommended by the WHO for TB diagnosis, demonstrates high sensitivity and specificity for the detection of TB and rifampin resistance. Its utility is limited, however, in certain cases of extra pulmonary TB (EPTB) [11, 12].

Recently, the emergence of metagenomic next-generation sequencing (mNGS) technology has heralded a new era in diagnostic capabilities. This comprehensive and hypothesis-free test offers unprecedented advantages in pathogen detection [13]. mNGS directly extracts and sequences all hereditary material fragments (DNA or RNA) from clinical samples, independently and simultaneously. Its wide-ranging application across various clinical specimens has led to its increasing utilization [14]. Studies have demonstrated that mNGS significantly outperforms traditional culture methods in terms of sensitivity and specificity for detecting pathogenic bacteria [15]. Nevertheless, the diagnostic efficacy of mNGS for detecting MTB DNA in PTB remains controversial [16, 17]. Our study aims to evaluate and compare the diagnostic accuracy of mNGS with other diagnostic methods for the diagnosis of PTB.

## Materials and methods

### Design and search strategy

A diagnostic test accuracy systematic review and meta-analysis were conducted. The study protocol was registered with PROSPERO, protocol number CRD42023427586. A comprehensive search was performed in PubMed (MEDLINE), Cochrane, Web of Science, and EMBASE databases. The search terms included #1 (tuberculosis); #2 (TB); #3 (*Mycobacterium tuberculosis*); #4 (lung); #5 (pulmonary); #6 (Metagenomic Next-Generation Sequencing); #7 (mNGS); #8 (Sequencing), and the search strategy were [(#1 OR #2 OR #3) AND (#4 OR #5) AND (#6 OR #7) AND (#8)]. The literature search was independently conducted by three authors (Y.Y, Y.N., and G.S.).

### Inclusion and exclusion criteria

Studies were included in the meta-analysis if they met the following criteria: (1) the study population consisted of patients with PTB, with at least one of the following sample types: BALF, sputum, lung biopsy tissue, or any other pulmonary samples; (2) diagnostic accuracy measures (true positive (TP), false negative (FN), true negative (TN), and false positive (FP)) were provided; (3) mNGS was one of the diagnostic methods used; and (4) sensitivity and specificity were reported as the main outcomes. Preclinical studies, editorials, review articles, commentaries, conference abstracts, and book chapters were excluded. Microbiologically confirmed TB cases were defined as those with MTB culture-positive or Xpert-MTB/RIF-positive results. Clinically diagnosed TB cases were those without microbiological evidence but with confirmed responsiveness to anti-TB treatment after 1 month of follow-up, in combination with clinical manifestations and imaging findings.

### Data extraction

Data were extracted from the individual studies by three authors (Y.Y, Y.N., and G.S.) and included the following: first author’s name, publication year, country of study, TP, FP, FN, and TN values for the assay, sample type, research type, number of patients, sequencing methods, sequencing conditions, and diagnostic methods. Disagreements were resolved through discussion among the authors.

### Statistical analysis

Sensitivity and specificity values were pooled using either a random-effects model or a fixed-effects model; 95% confidence intervals (CIs) were calculated to compare the diagnostic accuracy of mNGS with other detection methods. Statistical significance was set at* P* < 0.05*.* Heterogeneity was assessed using the* I*^2^statistic, with *I*^2^ ≤ 25% considered low and *I*^2^ ≥ 75% considered high. Receiver operating characteristic (ROC) curves were plotted for studies reporting both sensitivity and specificity. All statistical analyses were performed using R software version 4.1.0 (http://www.R-project.org) and Stata software (version 17.0).

### Quality score and the risk of bias assessment

Study quality was independently assessed by three reviewers using a revised version of the Quality Assessment of Diagnostic Accuracy Studies (QUADAS-2) tool [18]. Discrepancies between reviewers were resolved through discussion. The risk of bias and applicability of the included studies were evaluated according to the QUADAS-2 criteria.

## Results

### Characteristics of the studies

**Fig. 1:**
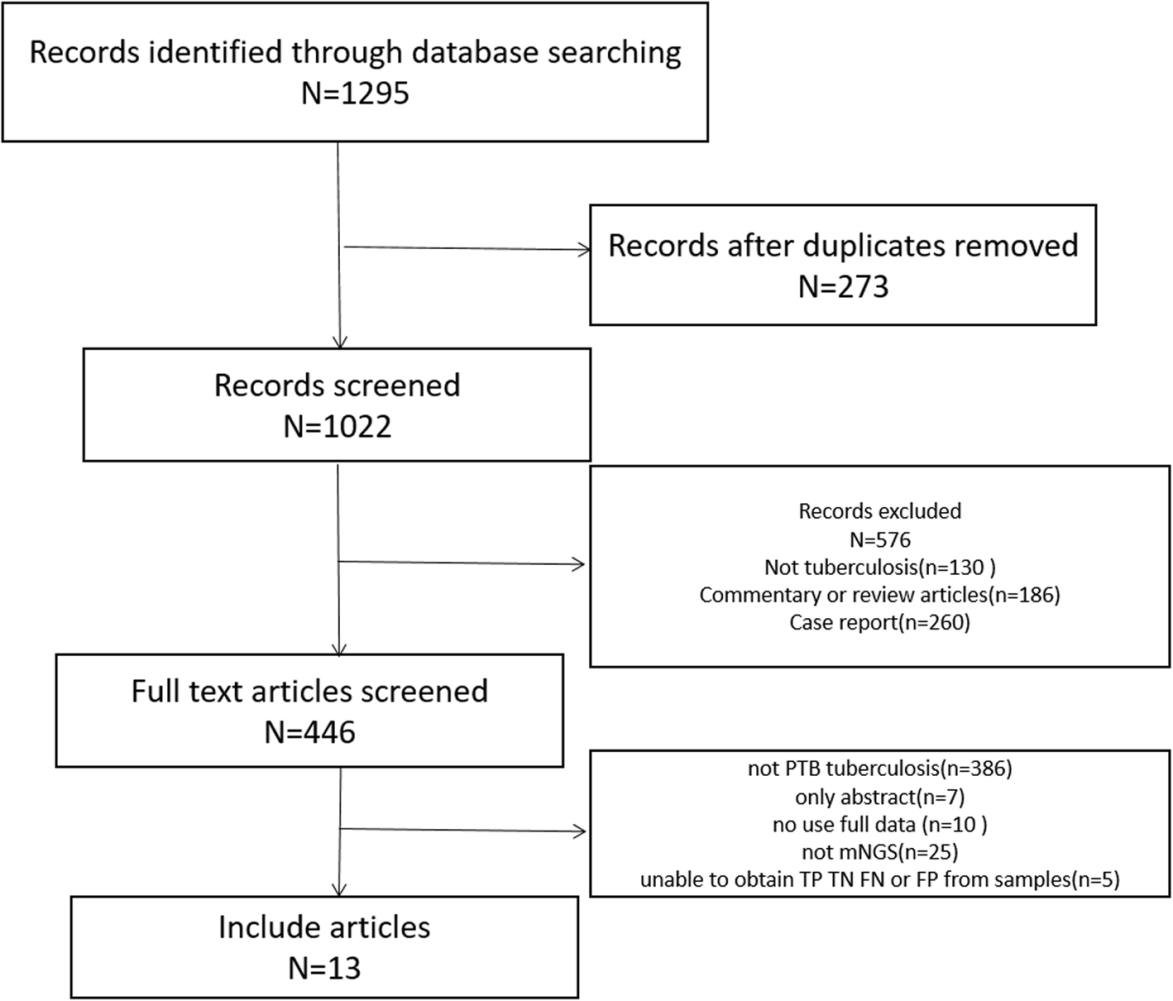
Flow chart of literature retrieval

Utilizing our search strategy, we identified 1295 candidate articles from relevant databases. Of these, 1282 articles did not meet our inclusion criteria and were excluded. Consequently, this meta-analysis encompassed 13 publications that met our criteria, spanning from 2019 to 2024 [16, 17, 19–29]. According to the PRISMA flowchart (Fig. 1), the included articles comprised nine retrospective and four prospective studies, exclusively conducted in China. The principal characteristics of the studies incorporated in this meta-analysis are detailed in Table 1.

**Table 1:** Summary of study characteristics

Author	Year	Country	Number of patients	Diagnostic methods	Sample type	TP	FP	FN	TN	Type of research	Sequencing methods	Sequencing conditions
Zhou X [19]	2019	China	105	Mycobacteria culture Xpert mNGS Xpert and mNGS	BALF/sputum/lung biopsy tissue	7/1	0/0	4/1	11/2	Prospective	DNA/ RNA-Seq	BGISEQ
Jin W T [17]	2020	China	820	Mycobacteria culture mNGS	BALF/ sputum/ lung biopsy tissue	11/12/8	2/4/1	10/11/1	108/265/44	Retrospective	DNA-Seq	BGISEQ
Shi C L [20]	2020	China	110	Mycobacteria culture Xpert mNGS Xpert and mNGS	BALF/sputum	23	1	25	61	Prospective	DNA-Seq	Others
Chen P X [16]	2020	China	70	Mycobacteria culture Xpert mNGS	BALF/sputum/lung tissue	11/3	0/0	3/0	14/3	Prospective	DNA-Seq	BGISEQ
Liu X [21]	2020	China	311	Mycobacteria culture AFB Xpert mNGS	BALF	118	28	93	83	Retrospective	DNA-Seq	BGISEQ
Zhu N [22]	2021	China	107	Mycobacteria culture AFB T-SPOT mNGS	BALF/ lung biopsy tissue	29/12	1/1	3/2	45/14	Retrospective	DNA-Seq	BGISEQ
Jin X [24]	2022	China	246	Mycobacteria culture AFB T-SPOT Xpert mNGS	BALF	19	99	9	119	Retrospective	DNA/ RNA-Seq	BGISEQ
Xu P [25]	2022	China	94	AFB T-SPOT Xpert mNGS	BALF/Lung biopsy specimens	67	0	4	23	Retrospective	DNA-Seq	Others
Fu M [23]	2022	China	403	Sputum stain PPD test CT Xpert mNGS	BALF/ lung biopsy tissue	21/15	0/0	15/21	21/15	Retrospective	DNA-Seq	Others
Gao J [26]	2023	China	186	Mycobacteria culture Xpert mNGS AFB	BALF	30	0	8	148	Retrospective	DNA -Seq	BGISEQ
Zhang D [29]	2023	China	217	Mycobacteria culture Xpert mNGS AFB ddPCR	Total lung specimens	86	0	14	117	Retrospective	DNA -Seq	Others
Hao J [27]	2023	China	266	Mycobacteria culture mNGS AFB T-SPOT Smear	BALF	40	3	10	112	Prospective	DNA -Seq	Others
Liu Y [28]	2023	China	52	mNGS AFB T-SPOT	Total lung specimens	20	8	0	13	Retrospective	DNA -Seq	Others

Total lung specimens including BALF and/or lung biopsy tissue and/or sputum

*CT* computerized tomography, *PPD* positive purified protein derivative, *AFB* acid-fast bacilli

### Study quality

**Fig. 2:**
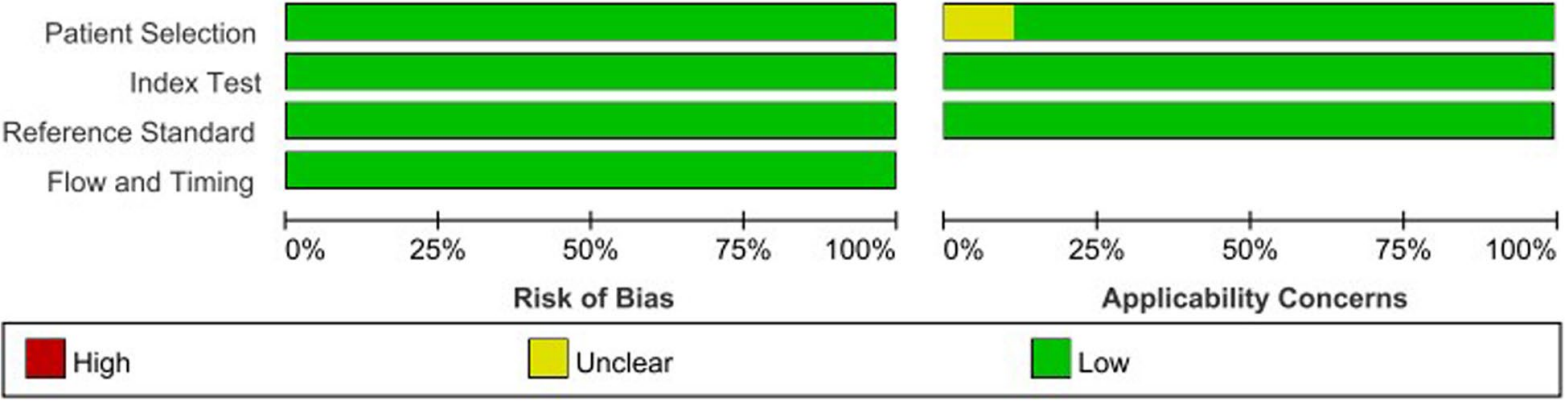
Categorized bar charts depicting risk of bias and applicability concerns in 13 included studies utilizing QUADAS-2. QUADAS-2 Quality Assessment of Diagnostic Accuracy Studies-2

The overall methodological quality of the included studies is presented in Fig. 2. The literature exhibited a low risk of bias in reference standard, patient selection, and flow and timing.

### Diagnostic accuracy of mNGS and other detection methods for PTB

**Fig. 3:**
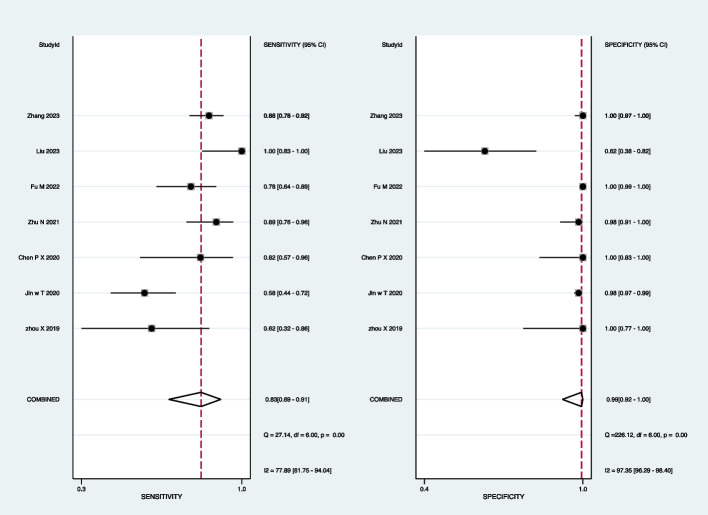
Forest plot displaying the sensitivity and specificity of mNGS across all pulmonary samples for the diagnosis of PTB

**Fig. 4:**
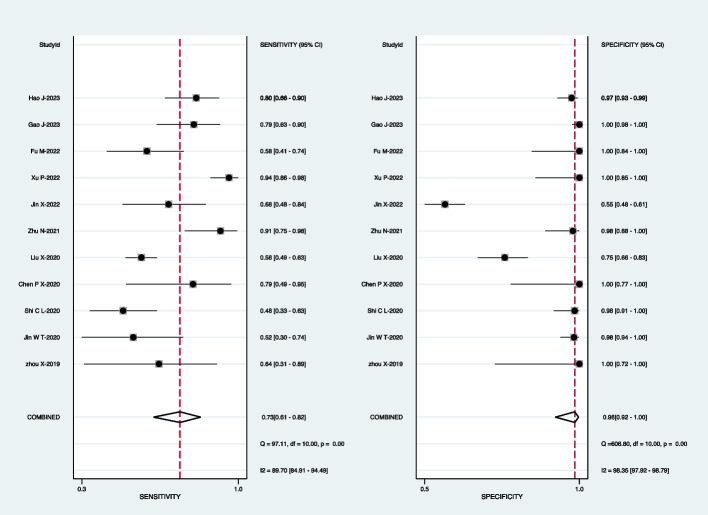
Forest plot illustrating the sensitivity and specificity of mNGS in BALF for the diagnosis of PTB

**Fig. 5:**
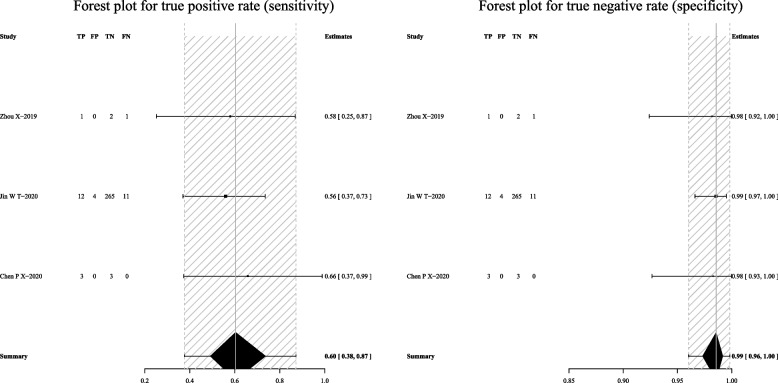
Forest plot depicting the sensitivity and specificity of mNGS in sputum samples for the diagnosis of PTB

**Fig. 6:**
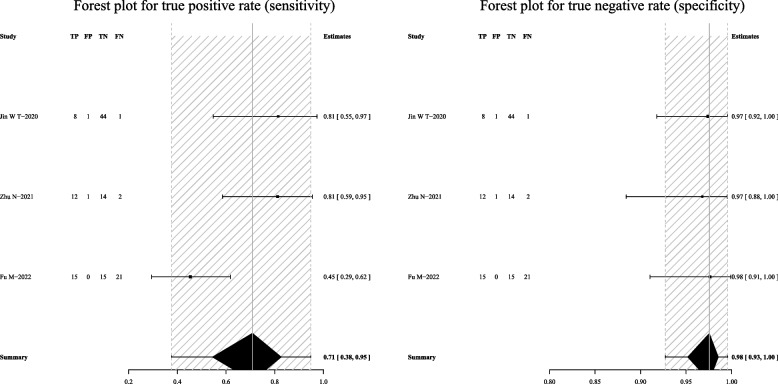
Forest plot showing the sensitivity and specificity of mNGS in the lung biopsy tissue for the diagnosis of PTB

For mNGS, the pooled sensitivity was 83% (95% CI: 69–91%) and the pooled specificity was 99% (95% CI: 92–100%) (Fig. 3). Subgroup analyses demonstrated that the pooled sensitivity was 73% (95% CI: 61–82%) and the pooled specificity was 98% (95% CI: 92–100%) in BALF (Fig. 4); the pooled sensitivity was 60% (95% CI: 38–87%) and the pooled specificity was 99% (95% CI: 96–100%) in the sputum (Fig. 5); the pooled sensitivity was 71% (95% CI: 38–95%) and the pooled specificity was 98% (95% CI: 93–100%) in the lung biopsy tissue (Fig. 6).

**Fig. 7:**
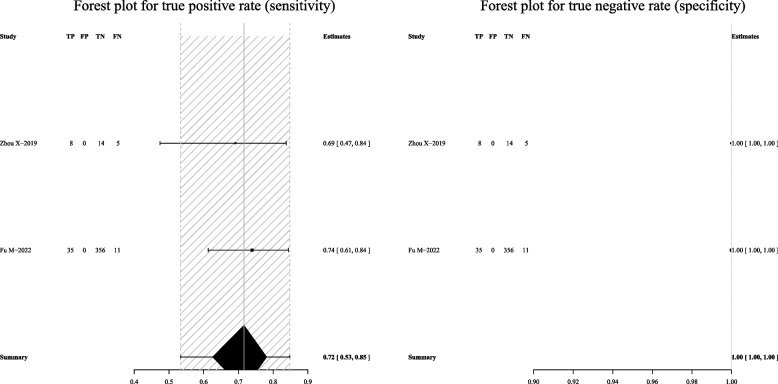
Forest plot outlining the sensitivity and specificity of Xpert-MTB/RIF across all pulmonary samples for the diagnosis of PTB

**Fig. 8:**
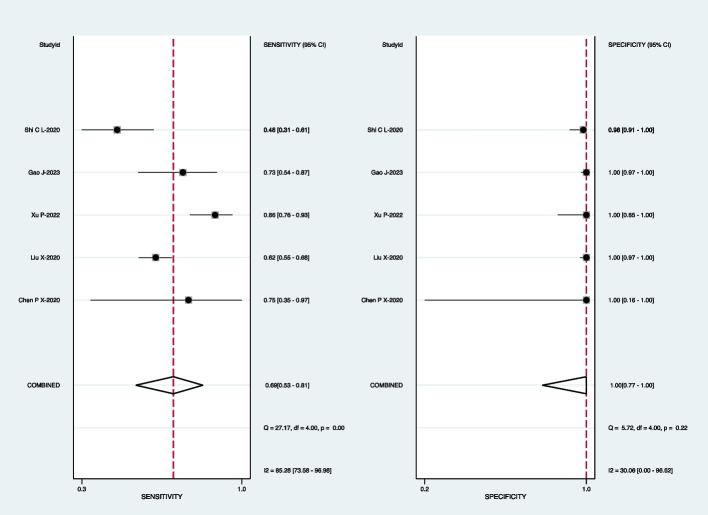
Forest plot demonstrating the sensitivity and specificity of Xpert-MTB/RIF in BALF for the diagnosis of PTB

For Xpert-MTB/RIF, the pooled sensitivity was 72% (95% CI: 53–85%) and the pooled specificity was 100% (95% CI: 100–100%) (Fig. 7). Subgroup analyses indicated that the pooled sensitivity was 69% (95% CI: 53–81%), and the pooled specificity was 100% (95% CI: 77–100%) in BALF (Fig. 8).

**Fig. 9:**
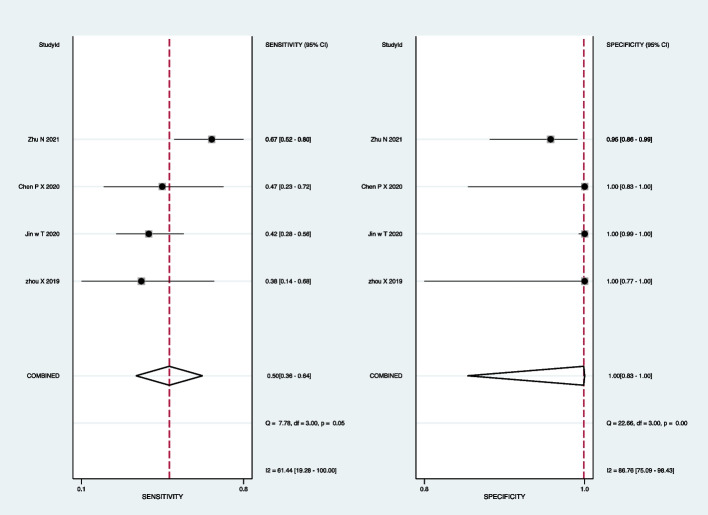
Forest plot representing the sensitivity and specificity of culture methods in all pulmonary samples for the diagnosis of PTB

**Fig. 10:**
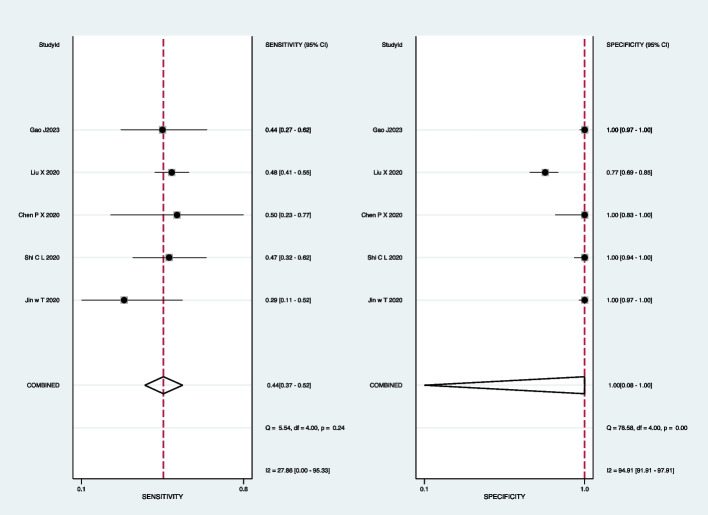
Forest plot highlighting the sensitivity and specificity of culture methods in BALF for the diagnosis of PTB

**Fig. 11:**
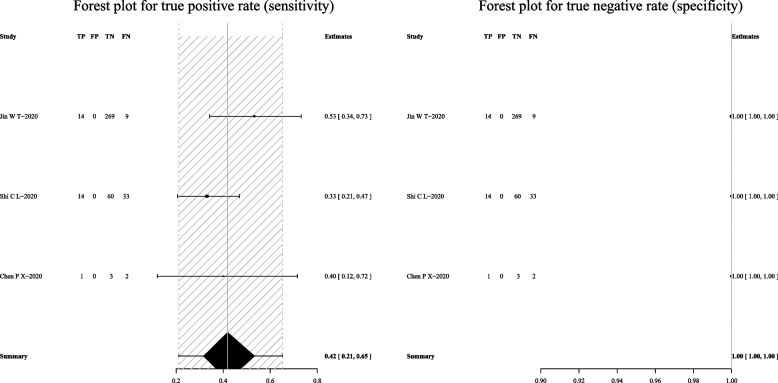
Forest plot portraying the sensitivity and specificity of culture methods in sputum samples for the diagnosis of PTB

For mycobacterial culture, the pooled sensitivity was 50% (95% CI: 36–64%) and the pooled specificity was 100% (95% CI: 83–100%) (Fig. 9). Subgroup analyses showed that the pooled sensitivity was 44% (95% CI: 37–52%), and the pooled specificity was 100% (95% CI: 8–100%) in BALF (Fig. 10); the pooled sensitivity was 42% (95% CI: 21–65%), and the pooled specificity was 100% (95% CI: 100–100%) in sputum (Fig. 11).

**Fig. 12:**
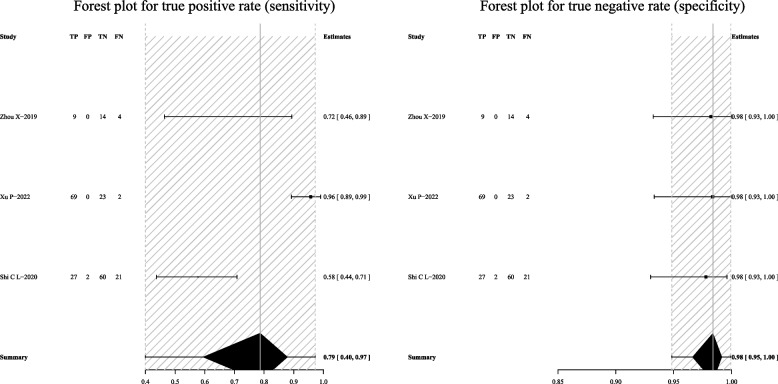
Forest plot exhibiting the sensitivity and specificity of mNGS combined with Xpert-MTB/RIF in all pulmonary samples for the diagnosis of PTB

When mNGS was combined with Xpert-MTB/RIF, the pooled sensitivity was 79% (95% CI: 40–97%) and the pooled specificity was 98% (95% CI: 95–100%) (Fig. 12).

**Fig. 13:**
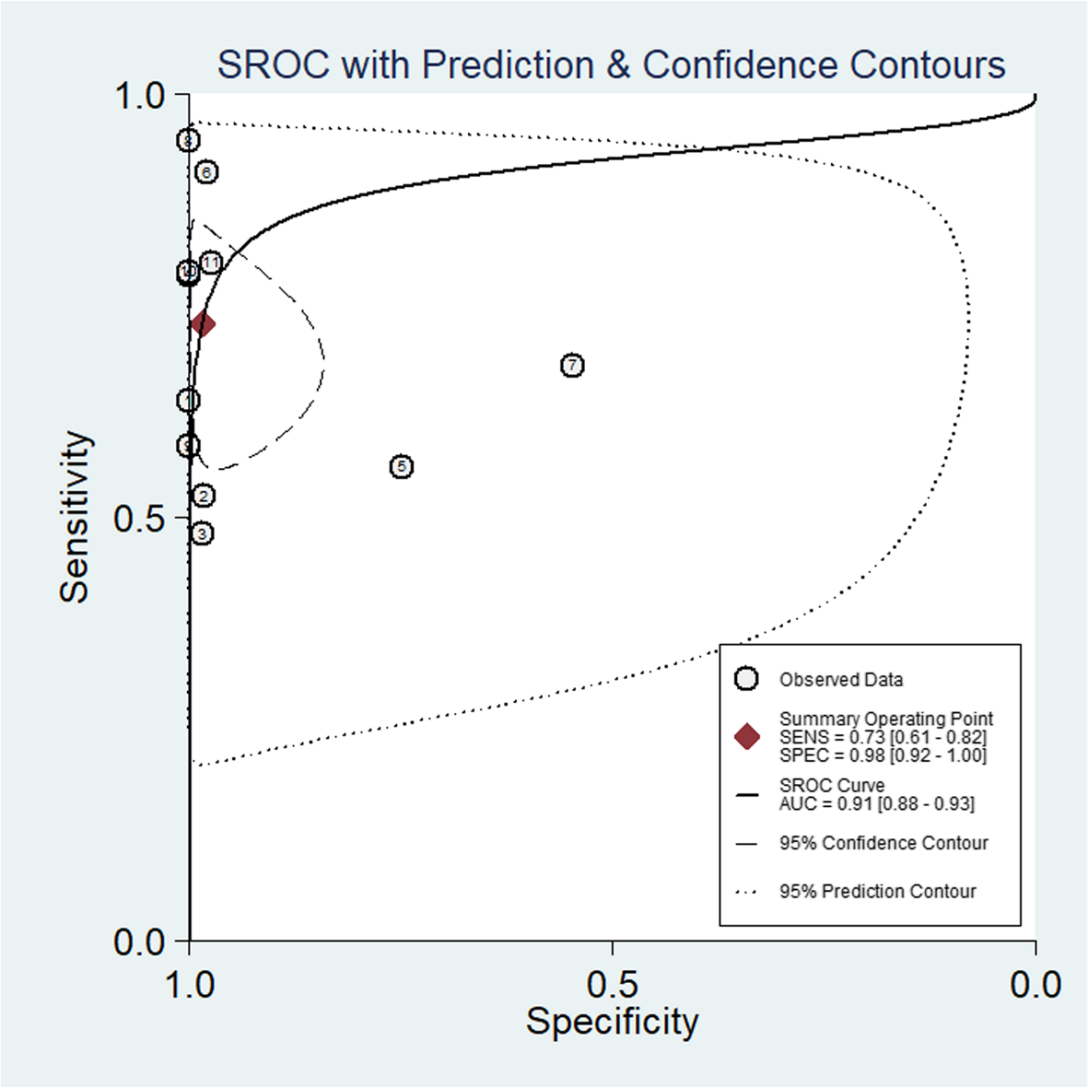
Summary receiver operating characteristic (SROC) plot encapsulating data from studies reporting Both the Sensitivity and Specificity of mNGS in BALF

The summary ROC (SROC) curves of these studies of mNGS in BALF exhibited a “shoulder-arm” shape with an area under the SROC curve (AUC) of 0.91 (Fig. 13). To further explore the heterogeneity among studies, we conducted meta-regression analyses. The type of study (prospective or retrospective), sequencing conditions (BGISEQ or others), homogenization, and sample pre-treatment (with or without bead-beating) were considered in the assay. Meta-regression analysis revealed that different types of studies (*P* < 0.00) and sample pre-treatment had a significant impact on the specificity of mNGS for BALF in PTB (*P* < 0.02). Similarly, the homogenization method had a significant effect on the sensitivity of mNGS for BALF in PTB (*P* < 0.00) (Table 2).

**Table 2:** Meta-regression analysis for different parameters

Parameter		Sensitivity (95% CI)	Specificity (95% CI)
Type of research	Prospective (4 studies)	69% (48–90%)	99% (97–100%)
	Retrospective (9 studies)	80% (69–90%)	98% (94–100%)
	Meta-regression *P*-value	0.13	0.00
Sequencing conditions	BGISEQ (7 studies)	72% (57–86%)	98% (93–100%)
	Others (6 studies)	82% (70–94%)	99% (97–100%)
	Meta-regression *P*-value	0.07	0.27
Homogenization	Yes (7 studies)	67% (53–80%)	99% (95–100%)
	No (6 studies)	86% (77–94%)	99% (95–100%)
	Meta-regression *P*-value	0.00	0.07
Sample pre-treatment	With bead-beating (6 studies)	82% (77–94%)	99% (97–100%)
	Without bead-beating (7 studies)	72% (57–86%)	98% (93–100%)
	Meta-regression *P*-value	0.87	0.02

## Discussion

In this meta-analysis, we aimed to compare the diagnostic performance of mNGS with Xpert-MTB/RIF, mycobacterial culture, and a combined approach of mNGS and Xpert-MTB/RIF for the detection of tuberculosis (TB) in pulmonary clinical specimens. Our findings revealed that the overall sensitivity of mNGS ranged from 60 to 83%, which was comparable to Xpert-MTB/RIF (69% to 72%) and superior to mycobacterial culture (42% to 50%). In contrast, the specificity of mNGS (98% to 99%) was similar to both Xpert-MTB/RIF (100%) and mycobacterial culture (100%). As Mycobacteria tuberculosis is not easy to detect, the sensitivity of the detection method is of utmost importance; thus, mNGS is often employed when conventional microbiological methods fail to identify the pathogen.

Our results demonstrated that mNGS exhibited a pooled sensitivity of 83% (95% CI: 69–91%) across all pulmonary samples, slightly higher than subgroup analyses for BALF (73%) and lung biopsy tissue (71%) and higher than sputum (60%). Notably, the specificity remained consistent across different pulmonary samples (98% to 99%), indicating its advantage in detecting a wide range of pulmonary samples, particularly BALF and lung biopsy tissue. These findings align with recent reports [17, 20, 22–24, 30] that highlight the remarkable diagnostic performance of mNGS in various samples from suspected TB patients. Regarding the detection of Xpert-MTB/RIF, the sensitivity of various pulmonary samples was observed to be 72%, slightly exceeding the sensitivity of BALF samples, which stood at 69%. Notably, the specificities in various pulmonary samples and BALF were both 100%. Although the sensitivity and specificity of Xpert-MTB/RIF detection were comparable to those of mNGS, which aligns with those reported by Zhou et al. [19], mNGS still offers an advantage over Xpert MTB/RIF in detecting the diversity of clinical samples. This advantage becomes particularly evident when certain pathogen infections cannot be definitively determined.

No surprise, the overall specificity of the mycobacterial culture was 100%. However, its sensitivity, especially in sputum samples, was very low. The overall sensitivity of the mycobacterial culture method ranged from 42 to 50%. Moreover, mycobacterial culture detection is known to be time-consuming. Thus, for sputum samples, mNGS can identify the pathogen more rapidly compared to traditional culture methods.

The studies enrolled [19, 20, 25] have provided data of combined mNGS with Xpert-MTB/RIF. The overall sensitivity and specificity of combined mNGS with Xpert-MTB/RIF were 79% and 98%, respectively. These values are comparable to those of mNGS and Xpert-MTB/RIF individually. These findings align with recent reports, indicating that when there is a high possibility of drug-resistant MTB, mNGS or mNGS combined with Xpert-MTB/RIF could be a better choice.

The AUC of the SROC for mNGS in BALF stood at 0.91 (95% CI: 0.88–0.93), indicating an exceptionally robust diagnostic performance of mNGS in BALF for PTB. However, it is worth noting that heterogeneity was observed across our results, affecting both sensitivity and specificity. To further investigate this heterogeneity among the included studies, meta-regression analyses were conducted. The results revealed that the heterogeneity of specificity was significantly associated with the type of study (prospective or retrospective) (*P* < 0.00) and the sample pretreatment methods employed (*P* < 0.02). Additionally, homogenization had a significant impact on reducing the heterogeneity of mNGS sensitivity in BALF for PTB (*P* < 0.00). However, it remains to be determined whether these factors truly influence the diagnostic accuracy of mNGS, and we urge caution in interpreting these findings. Furthermore, during the statistical analysis, we discovered that due to the challenges associated with DNA extraction and the low risk of contamination, most studies adopted a threshold of at least one taxon-specific read mapped to either the species or genus level to consider a result as MTB positive [13]. This implies that a single taxon-specific read serves as the positive threshold for MTB detection using mNGS, thus slight experiment error may change the mNGS result from negative to positive, or vice versa.

Currently, the utilization of mNGS technology faces some limitations, primarily attributed to the sequencing costs. But in our daily clinical practice, mNGS emerges as an excellent option. Its capabilities extend to the identification of a diverse range of pathogenic microorganisms, particularly useful in the differential diagnosis of suspected PTB with atypical radiologic performance. However, when considering the diagnostic tendencies and the associated costs, Xpert-MTB/RIF might still be a suitable choice for clinically typical PTB patients.

This meta-analysis still has some limitations. Firstly, the studies included in the analysis were not exclusively prospective randomized clinical trials; in fact, the majority were retrospective designs. Secondly, in addressing the sensitivity and specificity of mNGS, Xpert-MTB/RIF, and culture, we resorted to the R package “met bias” for analysis based on limited data from a few studies. This underscores the insufficiency of the available evidence. Thirdly, all the studies were conducted in China, and several of them suffered from a small sample size, thereby limiting their ability to accurately assess diagnostic precision. Consequently, the findings of this study should be interpreted with caution. Lastly, it is noteworthy that some of the results obtained from the subgroup analysis using the R package “met bias” exhibited no heterogeneity, which can complicate the interpretation and utility of the pooled effect estimate.

## Conclusions

This study demonstrated that mNGS had a comparable sensitivity and specificity to Xpert-MTB/RIF and a higher sensitivity than traditional mycobacterial culture methods. mNGS exhibited promising potential in detecting a diverse range of PTB clinical samples. The AUC analysis further corroborated the excellent diagnostic performance of mNGS for BALF. Despite the current limitations of mNGS technology, we think that ongoing advancements in this field will pave the way for novel diagnostic approaches in the future, thereby facilitating the diagnosis of PTB.

## Data Availability

The data supporting the results of this study are openly available from the reference listed in Table 1.

## Abbreviations

mNGS: Metagenomic next-generation sequencing
PTB: Pulmonary tuberculosis
BALF: Bronchoalveolar lavage fluid
NTM: Nontuberculous mycobacteria
MTB: Mycobacteria tuberculosis
AFB: Acid-fast bacilli
